# Room temperature spin diffusion in (110) GaAs/AlGaAs quantum wells

**DOI:** 10.1186/1556-276X-6-149

**Published:** 2011-02-16

**Authors:** Changcheng Hu, Huiqi Ye, Gang Wang, Haitao Tian, Wenxin Wang, Wenquan Wang, Baoli Liu, Xavier Marie

**Affiliations:** 1 School of Physics, Jilin University, Changchun 130021, PR China; 2 Beijing National Laboratory for Condensed Matter Physics, Institute of Physics, Chinese Academy of Sciences, P.O. Box 603, Beijing 100190, PR China; 3 INSA-CNRS-UPS; LPCNO, Université de Toulouse, 135 av. de Rangueil, 31077 Toulouse, France

## Abstract

Transient spin grating experiments are used to investigate the electron spin diffusion in intrinsic (110) GaAs/AlGaAs multiple quantum well at room temperature. The measured spin diffusion length of optically excited electrons is about 4 μm at low spin density. Increasing the carrier density yields both a decrease of the spin relaxation time and the spin diffusion coefficient *D*_s_.

## Introduction

The interest in the spin properties of carriers in semiconductors has increased dramatically in the past 10 years due to potential application in the field of spintronics [1,2]. The design of practical spintronic devices usually requires efficient spin injection in the semiconductor, long carrier spin lifetimes, and long spin transport/diffusion lengths [3-7].

One of the key parameters describing the properties of carrier spin transport in semiconductors is the spin diffusion coefficient *D*_s_, which is often assumed to be the same as charge diffusion coefficient *D*_c _[8]. A direct optical measurement of the electron spin diffusion coefficient can be performed by creating electron spin grating in time-resolved four-wave mixing experiments [9]. This powerful transient spin grating (TSG) technique was used recently to study the spin transport properties and determine the spin diffusion coefficient *D*_s _[9-11]. In particular it was demonstrated theoretically and experimentally that the spin diffusion coefficient *D*_s _in *n*-doped (100)-grown GaAs quantum wells can be smaller than the charge diffusion coefficient *D*_c _due to Coulomb interaction among the electrons (the so-called Spin Coulomb Drag effect) [10,12]. In these (100)-grown GaAs quantum wells, the electron spin lifetime is of the order of 100 ps at room temperature (RT) due to very efficient D'yakonov-Perel (DP) spin relaxation mechanism [13]. In the classical two-component drift-diffusion model [14], the spin diffusion length *L*_s _is determined by the spin lifetime $\tau_{\text{s}}^{*}$ and the spin diffusion coefficient *D*_s _through $L_{\text{s}}=\sqrt{D_{\text{s}}\tau_{\text{s}}^{*}}$. As a consequence, the spin diffusion length *L*_s _at RT is smaller than 1 μm, limited by the short spin lifetime [10]. In (110)-grown GaAs/AlGaAs QW, the DP spin relaxation mechanism is not efficient for electron spins parallel to the growth direction because the spin orientation of electrons is parallel to the direction of effective magnetic field induced by spin-orbit coupling [15]. Spin relaxation times longer than 1 ns at RT in (110) GaAs QW have indeed been measured [16]. Long electron spin diffusion lengths can thus be expected at high temperature in these structures. In this report, the electron spin diffusion is measured by the TSG technique with heterodyne detection in (110) GaAs/AlGaAs QWs at RT. We find that the spin diffusion length *L*_s _is about 4 μm at low carrier density. We also demonstrate that the spin diffusion coefficient *D*_s _decreases when the carrier density increases.

## Experimental procedure

**Figure 1:**
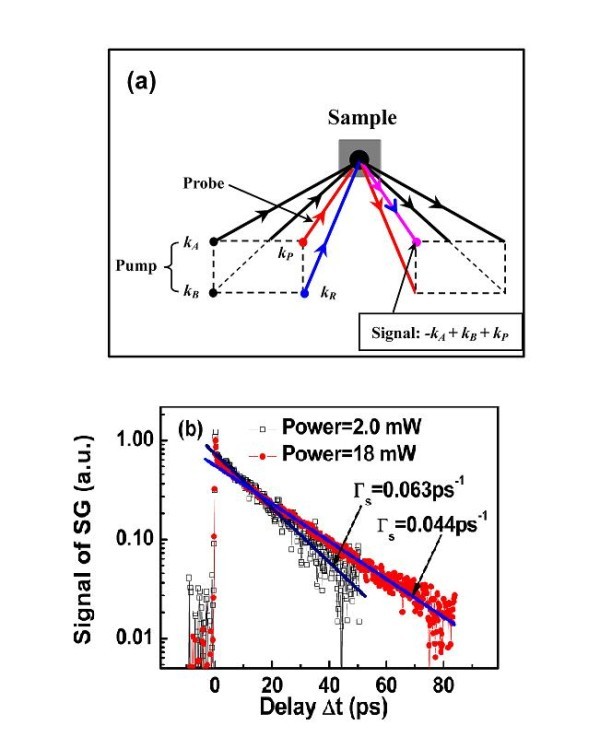
**Schematic drawing of TSG setup and TSG signals**. (a) *k*_A _and *k*_B _represent both the pump beams, *k*_P _is the probe beam, and *k*_R _is the reference beam. **(b) **TSG signal as a function of delay time at room temperature for two excitation powers: 2 and 18 mW.

The investigated sample was grown on (110)-oriented semi-insulating GaAs substrate by molecular beam epitaxy. It consists of 20 planes of 8 nm thick GaAs QW with symmetric 27 nm Al_0.28_Ga_0.72_As barriers on both sides. The sample is nominally undoped. All the measurements are performed at RT. In the spin grating experiment, the laser pulses are generated by a mode-locked Ti:sapphire laser with 120 fs pulse duration and 76 MHz repetition frequency and split into primary pump and probe beams. The center wavelength is set to 830 nm to get the maximum signal of Kerr rotation through the standard time-resolved Kerr rotation technique [17]. Both pump and probe beams are focused on a phase mask with a period *d*. The phase mask splits each of the primary beams by diffraction into the *m *= ± 1 orders. The geometry of the spin grating experiment in the so-called box geometry is schematically presented in Figure 1a[18,19]. For orthogonal-linearly polarized pumps, the net polarization alternates between right and left circular polarization across the excitation spot while the total intensity of the incident light is uniform [9]. The period Λ of the TSG is simply: $\Lambda =\frac{d}{2}\cdot \frac{f_{2}}{f_{1}}$, where *f*_1 _and *f*_2 _are the focal lengths of two spherical mirrors. In our setup, the focal length of the first spherical mirror is fixed at *f*_1 _= 30.4 cm. The focal length *f*_2 _of the second spherical mirror can be changed to get a fine tuning of the period Λ. The spot sizes of both pump and probe beams are around 90 μm.

According to the optical interband selection rules, this interference pattern will generate a periodical spin density in the sample. The delayed probe beam, diffracted from the grating, is monitored as a function of the delay time between the pump and the probe. In order to enhance the signal-to-noise ratio, a reference beam is incident on the sample and its reflected beam is automatically collinear with the refracted probe beam. In this configuration, the spin grating signal (i.e., proportional to the electric field of the diffracted probe beam) is simply given by:

(1) $$I_{\text{SG}}=A\exp(-\Gamma_{\text{s}}\Delta t)$$

where *A *is a constant, Γ_s _is the decay rate of the spin grating, and Δ*t *is the delay time between pump and probe beams.

## Results and discussion

Figure 1b presents the signal of TSGs as a function of the time delay for two typical pump powers, 2 and 18 mW, respectively. The wave vector *q *of the spin grating is equal to $q=\frac{2\pi }{\Lambda }=\text{2}.\text{51}\times {\text{10}}^{\text{4}}\;{\text{cm}}^{-\text{1}}$. It is clear that both curves exhibit different mono-exponential decays. Using equation (1), we find Γ_s _= 0.063 and 0.044 ps^-1 ^for the pump powers 2 and 18 mW, respectively.

**Figure 2:**
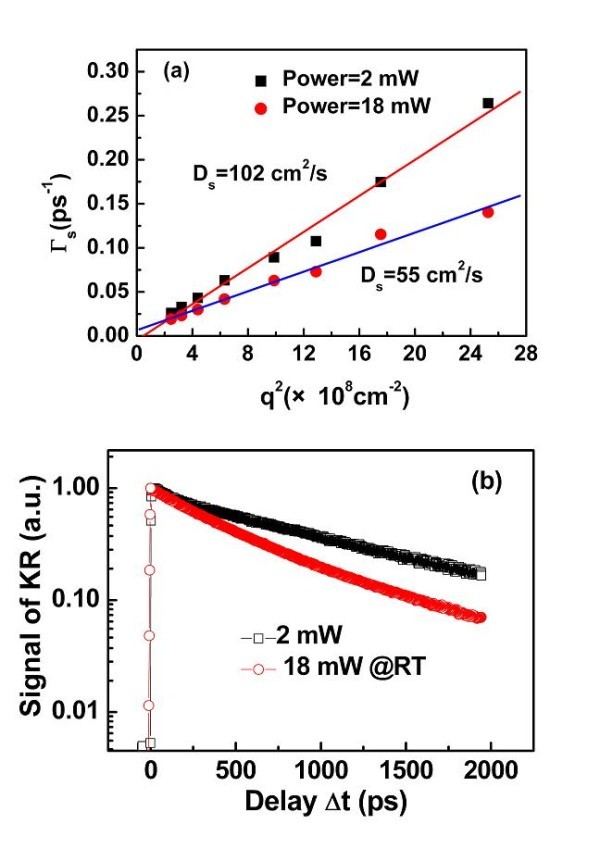
**Spin diffusion coefficient and spin dynamics for two different powers**. **(a) **Decay rate of spin grating as a function of *q*^2 ^for two excitation powers: 2 and 18mW. **(b) **Kerr rotation dynamics obtained from homogenous spin excitation.

In the diffusion regime, the SG decay rate writes [8,9]:

(2) $$\Gamma_{\text{s}}=D_{\text{s}}q^{2}+\frac{1}{\tau_{\text{s}}^{*}}$$

where *D*_s _is the spin diffusion coefficient, *q *is the spin grating wave vector, and $\tau_{\text{s}}^{*}$ is the spin lifetime which includes the effect of both the electron spin relaxation time τ_s _and the recombination time τ_r, _as expressed by $\frac{1}{\tau_{\text{s}}^{*}}=\frac{1}{\tau_{\text{s}}}+\frac{1}{\tau_{\text{r}}}$. To separate the effects of spin diffusion and spin relaxation, the grating decay rate is measured as a function of the grating wave vector *q *by changing the phase mask with different periods (*d *= 5, 6, 7, and 8 μm) and/or the second spherical mirror with different focus lengths (*f*_2 _= 15.2 and 30.4 cm). Figure 2a shows the grating decay rate as a function of *q*^2 ^for two excitation powers. Each set of data points can be fitted linearly, yielding the spin diffusion coefficient *D*_s_. At low excitation power of 2 mW, which corresponds to an optical intensity of 30W/cm^2^, we find *D*_s _= ~102 cm^2^/s. This value is in good agreement with the values obtained by other groups in (110)-grown GaAs/AlGaAs QWs at RT [8,20]. It is also very close to the spin diffusion coefficient *D*_s _measured in (100)-grown GaAs/AlGaAs QWs at RT [9,10]. This result suggests that the spin diffusion coefficient *D*_s _does not depend critically on the spin-orbit coupling, which depends on the crystalline direction of the sample. Nevertheless, as shown in Figure 2a, it is very sensitive to the carrier density.

In order to obtain the spin diffusion length *L*_s_, the spin lifetime $\tau_{\text{s}}^{*}$ is measured independently by time-resolved Kerr rotation [17]. The excitation powers are the same as the ones used in the measurement of TSG. Figure 2b presents the Kerr rotation dynamics for two excitation powers. The spin lifetimes $\tau_{\text{s}}^{*}$ are extracted by mono-exponential fits, which yield $\tau_{\text{s}}^{*}$ ~1220 ps and $\tau_{\text{s}}^{*}$ ~880 ps with excitation powers of 2 and 18 mW, respectively. As expected for (110)-grown QWs, the spin lifetimes for both excitation powers are much longer than the ones ($\tau_{\text{s}}^{*}$ ~ 50-100 ps) measured in (100)-grown GaAs/AlGaAs QWs at RT [9]. By combining the *D*_s _measurement obtained with the spin grating technique and the electron spin lifetime probed by the Kerr rotation experiment, we find that the spin diffusion length decreases from *L*_s _~ 3.5 μm down to 2.2 μm when the excitation power increases from 2 to 18 mW. To the best of our knowledge, these values are the longest electron spin diffusion lengths reported at room temperature in inorganic semiconductors.

In order to get further insights on this power dependence, we also measured the charge diffusion coefficient *D*_c _with a concentration grating technique for different pump powers. We find that *D*_c _remains constant with a typical value *D*_c _~ 12.5 cm^2^/s (data not shown here). This value is in good agreement with previous studies performed in non-intentionally doped (100)-grown GaAs QWs which demonstrate that the concentration grating experiments are governed by the hole diffusion [9].

**Figure 3:**
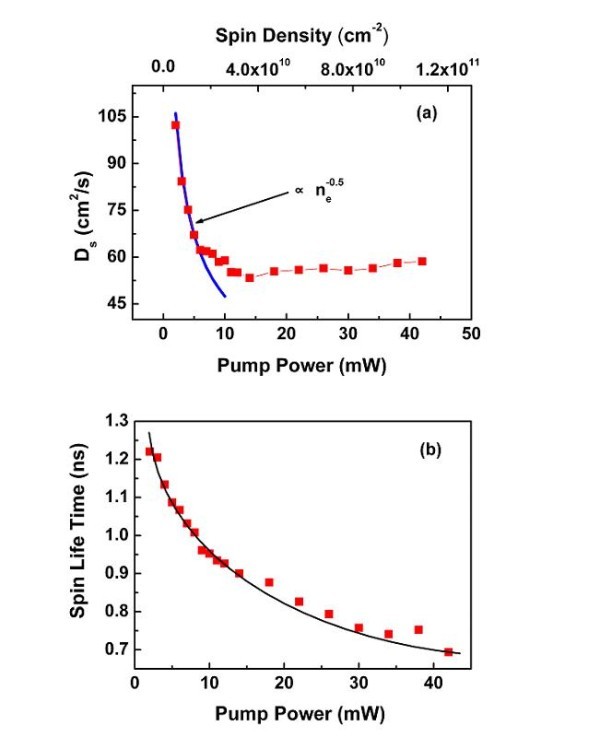
**Power-dependence spin diffusion coefficient and spin lifetime**. **(a) **Spin diffusion coefficient *D*_s _versus pump power, i.e., spin density; the blue line is a simple fit according to $\tau_{\text{p}}\propto n_{\text{ex}}^{-0.5}$. **(b) **Pump power-dependent spin lifetime through Kerr rotation measurement with a fixed probe power of 0.2 mW.

Our spin diffusion coefficient results obtained at RT on (110) QWs contrast with the previous measurements of the carrier density dependence of the spin diffusion obtained at low temperature in *n*-doped bulk GaAs or (100) quantum wells [11,21]. In *n*-doped QWs, Carter et al. observed that *D*_s _*increases *by increasing the density of the optically excited carriers. This increase of the electron spin diffusion coefficient was interpreted in terms of heating of the excess electrons due to relaxation of energetic optically excited carriers. Remarkably, in non-intentionally doped GaAs (110)-grown QWs, we observe at room temperature the opposite behavior. As displayed in Figure 3a, the spin diffusion coefficient *D*_s _*decreases *abruptly for a pump power varying between 2 and 10 mW, and then remains almost coefficient up to 40 mW. In the same power range the spin lifetime (Figure 3b) has a different power dependence: it decreases monotonously as already observed by different groups, due to electron spin relaxation enhancement by the electron-hole exchange interaction [16]. Since the sample was undoped, we can equate the electron spin diffusion coefficient *D*_s _to the electron charge diffusion coefficient *D*_e_. The spin diffusion coefficient *D*_s _can thus be written [22]:

(3) $$D_{\text{s}}=D_{\text{e}}=\frac{<v^{2}>\tau_{\text{p}}}{2}$$

where <*v*^2^> is the mean square velocity of electrons and τ_p _is the momentum relaxation time. In a very simple approach, <*v*^2^> in a QW can be approximated by $<v^{\text{2}}>=\sqrt{2k_{\text{B}}T/m_{\text{e}}^{*}}$. The momentum relaxation τ_p _is strongly dependent on the density of photogenerated electrons *n*_e_, with a typical power law $\tau_{\text{p}}\propto n_{\text{e}}^{-0.5}$[23]. In the low density regime below 2.5 × 10^10 ^cm^-2^, which corresponds to a pump power of 10 mW, the experimental data are well fitted by this power law as shown by the blue line in Figure 3a. In the high density regime above 2.5 × 10^10 ^cm^-2^, the spin diffusion coefficient is almost constant and the density dependence can no more be interpreted by the simple power law. In this density range, the above discussion is clearly oversimplified and we hope that these experimental results will stimulate theoretical investigations to elucidate the origin of the carrier density dependence of the spin diffusion coefficient.

## Conclusions

We have measured optically the spin diffusion coefficient *D*_s _in non-intentionally doped GaAs/AlGaAs (110) QWs at room temperature for different excitation powers. Under low excitation, the electron spin diffusion length *L*_s _is around 4 μm; to the best of our knowledge, this is the largest reported value at *T *= 300 K in III-V semiconductors. We also show that the spin diffusion coefficient of optically excited electrons decreases when the excitation density increases. These results could be useful to understand the spin transport properties in semiconductor structures, and possibly control/manipulate the spin transport by varying the excitation condition.

## Abbreviations

DP: D'yakonov-Perel; TSG: transient spin grating.

## Competing interests

The authors declare that they have no competing interests.

## Authors' contributions

CC, BL conceived and designed the experiments. CC, HQ carried out the experiments with contribution from GW and WQW. WXW and HT provided the samples. BL and XM supervised the work. CC, BL and XM wrote the manuscript. All authors read and approved the final manuscript.

## Open Access

This article is distributed under the terms of the Creative Commons Attribution Noncommercial License which permits any noncommercial use, distribution, and reproduction in any medium, provided the original author(s) and source are credited.
